# M protein of subacute sclerosing panencephalitis virus, synergistically with the F protein, plays a crucial role in viral neuropathogenicity

**DOI:** 10.1099/jgv.0.001682

**Published:** 2021-10-13

**Authors:** Yuto Satoh, Kurara Higuchi, Daichi Nishikawa, Hiroshi Wakimoto, Miho Konami, Kento Sakamoto, Yoshinori Kitagawa, Bin Gotoh, Da-Peng Jiang, Hak Hotta, Masae Itoh

**Affiliations:** ^1^​ Department of Microbiology, Faculty of Bio-Science, Nagahama Institute of Bio-Science and Technology, Nagahama, Shiga, Japan; ^2^​ Division of Microbiology and Infectious Diseases, Department of Pathology, Shiga University of Medical Science, Otsu, Shiga, Japan; ^3^​ Graduate School of Medicine, Kobe University, Kobe, Hyogo, Japan; ^†^​ Present address: Faculty of Clinical Nutrition and Dietetics, Konan Women’s University, Kobe, Hyogo, Japan

**Keywords:** measles virus, SSPE, neuropathogenicity, matrix protein, fusion protein

## Abstract

Subacute sclerosing panencephalitis (SSPE) is a rare fatal neurodegenerative disease caused by a measles virus (MV) variant, SSPE virus, that accumulates mutations during long-term persistent infection of the central nervous system (CNS). Clusters of mutations identified around the matrix (M) protein in many SSPE viruses suppress productive infectious particle release and accelerate cell–cell fusion, which are features of SSPE viruses. It was reported, however, that these defects of M protein function might not be correlated directly with promotion of neurovirulence, although they might enable establishment of persistent infection. Neuropathogenicity is closely related to the character of the viral fusion (F) protein, and amino acid substitution(s) in the F protein of some SSPE viruses confers F protein hyperfusogenicity, facilitating viral propagation in the CNS through cell–cell fusion and leading to neurovirulence. The F protein of an SSPE virus Kobe-1 strain, however, displayed only moderately enhanced fusion activity and required additional mutations in the M protein for neuropathogenicity in mice. We demonstrated here the mechanism for the M protein of the Kobe-1 strain supporting the fusion activity of the F protein and cooperatively inducing neurovirulence, even though each protein, independently, has no effect on virulence. The occurrence of SSPE has been estimated recently as one in several thousand in children who acquired measles under the age of 5 years, markedly higher than reported previously. The probability of a specific mutation (or mutations) occurring in the F protein conferring hyperfusogenicity and neuropathogenicity might not be sufficient to explain the high frequency of SSPE. The induction of neurovirulence by M protein synergistically with moderately fusogenic F protein could account for the high frequency of SSPE.

## Introduction

Measles virus (MV) is the causative agent of the highly contagious acute disease measles, with characteristic symptoms such as high fever, cough and maculopapular rash [1, 2]. Entering the host by the respiratory route, MV first infects dendritic cells and alveolar macrophages in the respiratory tract and spreads via the bloodstream to lymphoid organs throughout the body using signalling lymphocyte activation molecule (SLAM, also called CD150) expressed on immune cells as a cellular receptor [3, 4]. In the late stage of infection, MV infects respiratory epithelial cells using nectin 4 as a receptor [5, 6], followed by shedding of progeny viral particles via coughing and sneezing to transmit to susceptible new hosts [2, 7, 8]. Very rarely, MV may break into the central nervous system (CNS) and persist independently of SLAM and nectin 4 because neither is expressed in the human CNS [9, 10], causing a rare fatal neurodegenerative disease, subacute sclerosing panencephalitis (SSPE) [1]. SSPE usually occurs 6 to 8 years on average after contraction of acute measles, as a result of accumulated mutations in the viral genome.

MV, a member of the genus *Morbillivirus* in the family *Paramyxoviridae*, is an enveloped virus with a non-segmented negative-sense RNA genome encoding six proteins: nucleocapsid protein (N), phosphoprotein (P), matrix protein (M), fusion protein (F), haemagglutinin protein (H) and large protein (L). The P gene also encodes the additional accessory proteins, V and C [1, 11]. Two envelope glycoproteins, the H and F proteins forming an H/F protein complex [12], have roles in receptor binding and membrane fusion, respectively, to introduce the ribonucleoprotein (RNP) complex, composed of the RNA genome and the N, P and L proteins, into the host cell by fusing the viral envelope with the plasma membrane (envelope fusion) and thus initiating infection [13–17]. The H and F proteins expressed on the surface of the infected cell also elicit fusion of the plasma membrane with that of adjacent cells, forming a multinuclear giant cell, a syncytium, to spread infection (cell–cell fusion) [1]. The M protein promotes formation of viral particles and plays an important role in the assembly of infectious viral particles by interacting with the RNP complex as well as the cytoplasmic tails of the H and F proteins and taking them into the viral particle [11, 18].

MV variants isolated from the brains of SSPE patients, known as SSPE virus, differ from wild-type MV and have specific characteristics: lack of infectious viral particle production, enhanced cell–cell fusion ability, and neurovirulence in rodents [19–23]. In the genome, clusters of mutations occur around the M gene in many SSPE viruses [24–28]. Recombination experiments in which the M gene of MV was replaced by that of an SSPE virus or was deleted from the genome revealed that abrogation of M protein function accounts for the lack of particle formation and the efficient cell–cell fusion ability of SSPE viruses, but not for neurotoxicity. However, M protein might be involved in the establishment of persistent infection in the brain [29, 30]. Recently, such mutations were identified in the ectodomain of the F protein, and enhanced its fusion activity, leading to the formation of syncytia of receptor (SLAM and/or nectin 4)-negative cells, a key characteristic related to the neuropathogenicity of SSPE viruses [31, 32].

The Kobe-1 strain of SSPE virus isolated from the brain of a 5-year-old patient only 6 weeks after the onset of SSPE possessed very few mutations from the wild-type MV field isolate prevalent in Japan and from which the Kobe-1 strain possibly originated [33]. It is likely that the Kobe-1 strain was isolated before additional accumulation of mutations and carries the minimum mutations required for the onset of SSPE. In this study, we identified the gene(s)/protein(s) responsible for its neurovirulence and found that the F protein of the Kobe-1 strain did not itself confer neurovirulence and that mutations in the M protein are indispensable for pathogenicity in suckling mice. The mechanism by which M protein and F protein promote the neurovirulence of the Kobe-1 strain is demonstrated, and the role of the M protein in neuropathogenicity is discussed.

## Results

### The M gene of the SSPE virus Kobe-1 strain is indispensable for neuropathogenicity

**Fig. 1.**
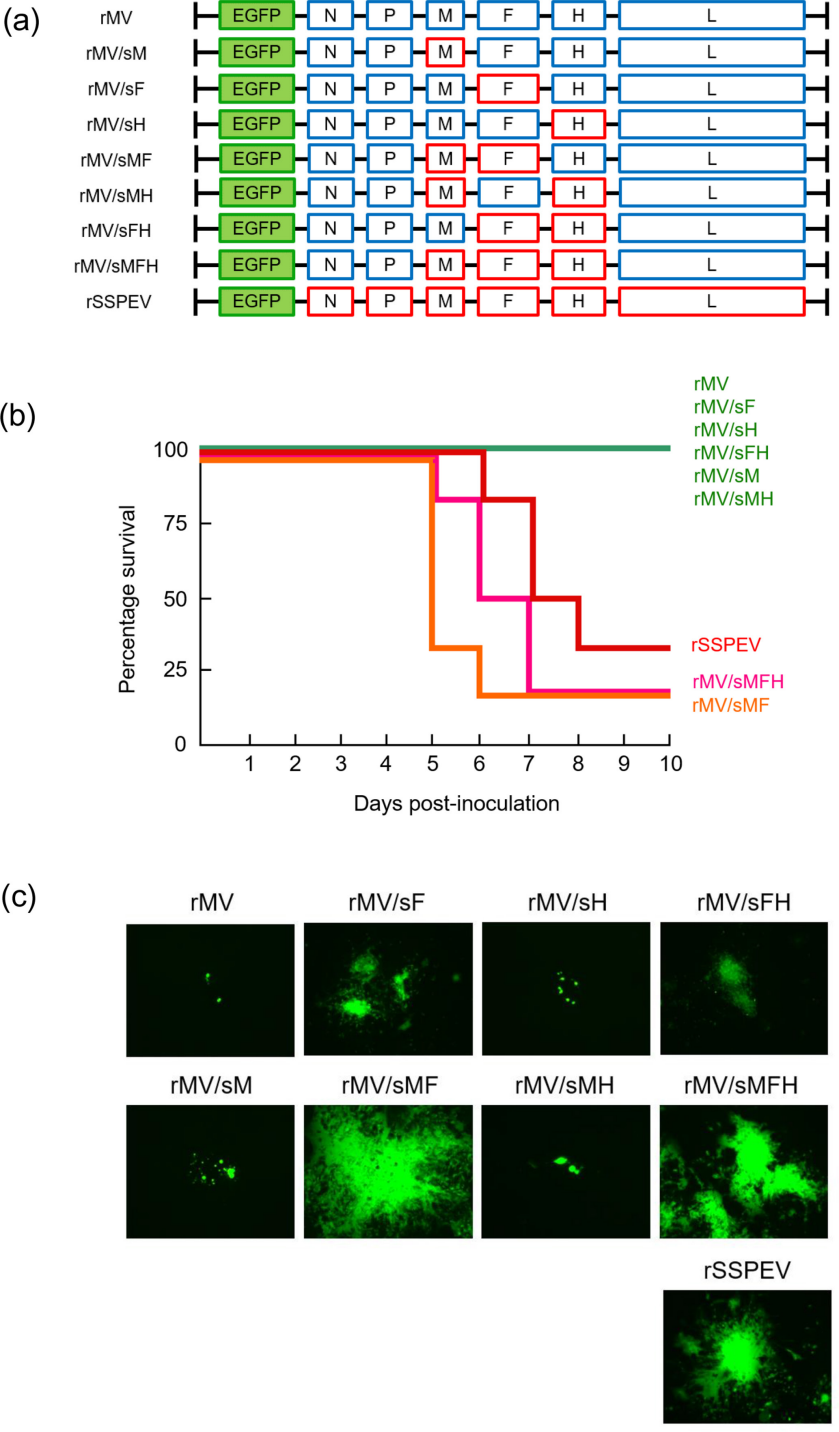
Requirement of the M gene for neurovirulence by the SSPE virus Kobe-1 strain. (**a**) Schematic diagram of the genomes of the EGFP-expressing recombinant chimeric viruses. The protein-coding regions from the measles virus ICB strain (rMV) are shown as blue boxes, and those from the SSPE virus Kobe-1 strain (rSSPEV) as red boxes. (**b**) Mortality of suckling mice intracerebrally inoculated with chimeric viruses. Six suckling mice were infected with 7×10^2^ p.f.u. of virus and monitored for 10 days. (**c**) Spread of infection in neuronal cells. SH-SY5Y cells were infected with chimeric virus at an m.o.i. of 0.001. After incubation at 37 °C for 7 days, EGFP-expressing infected cells were observed under a fluorescence microscope. Magniﬁcation, ×200.

Recent studies have identified the F protein, not the previously suggested M protein, of the SSPE viruses as the viral element responsible for neuropathogenicity [31, 32, 34]. To identify the viral gene(s)/protein(s) that cause neurovirulence of the SSPE virus Kobe-1 strain, the F gene of an EGFP-expressing recombinant MV ICB strain (rMV), the D3 genotype virus, which is close to the parental MV of the Kobe-1 strain, was replaced by that of the Kobe-1 strain (Fig. 1a). The recovered virus rMV/sF, however, showed no lethality when inoculated in the brain of suckling mice, like other chimeric viruses such as rMV/sM and rMV/sH, the recombinant MV ICB strains possessing the M and H genes of the SSPE virus Kobe-1 strain, respectively (Fig. 1b). Next, we introduced the M, F and H genes of the Kobe-1 strain in combination into the genome of the MV ICB strain (Fig. 1a). As shown in Fig. 1b, the chimeric viruses possessing the M and F genes of the SSPE virus Kobe-1 strain, rMV/sMF, were highly lethal and the rMV/sMFH with the M, F and H genes of the Kobe-1 strain killed mice slightly more slowly. By contrast, chimeric virus carrying the F and H genes, or the M and H genes of the Kobe-1 strain, rMV/sFH or rMV/sMH, did not kill mice. These results demonstrate that the M gene of the SSPE virus Kobe-1 strain is required in addition to the F gene for neurovirulence in mice, and that the H gene of the SSPE Kobe-1 strain might attenuate virulence. The recombinant SSPE virus Kobe-1 strain (rSSPEV) killed mice more slowly than the chimeric virus rMV/sMFH, suggesting that the Kobe-1 strain carries other gene(s) than the H gene that attenuate neurovirulence.

To examine the propagation of the neurovirulent viruses carrying the genes of the SSPE virus Kobe-1 strain in human neuronal cells, SH-SY5Y neuroblastoma cells were infected with the chimeric viruses. rMV/sF spread slightly more efficiently than rMV, which was significantly facilitated by the introduction of the M gene of the Kobe-1 strain (rMV/sMF) (Fig. 1c). When rMV/sMFH was compared with rMV/sFH, rMV/sMFH spread more efficiently, indicating that the Kobe-1 M gene strongly promotes viral growth in neuronal cells. By contrast, replacement of the M gene of the viruses with the MV ICB F gene (e.g. rMV and rMV/sH) by the M gene of the Kobe-1 strain enhanced viral replication slightly (rMV/sM and rMV/sMH, respectively). rMV/sFH and rMV/sMFH replicated less efficiently than rMV/sF and rMV/sMF, respectively, suggesting that the H gene of the SSPE Kobe-1 strain suppresses viral growth in neuronal cells. The neurovirulence of the chimeric viruses in mice is correlated with their ability to grow in neuronal cells: rMV/sMF, rMV/sMFH and rSSPEV (in that order) spread efficiently in SH-SY5Y cells. The F gene of the SSPE virus Kobe-1 strain encodes an F protein with nine amino acid substitutions as well as a shortened cytoplasmic tail, and the M gene carries three amino acid substitutions compared with the MV ICB strain, respectively [33]. These results indicate that some mutations in the F protein and M protein of the SSPE Kobe-1 strain are indispensable for viral propagation in the brain and neurovirulence.

### The M protein of the SSPE virus Kobe-1 strain significantly enhances the fusion activity of Kobe-1 F protein

**Fig. 2.**
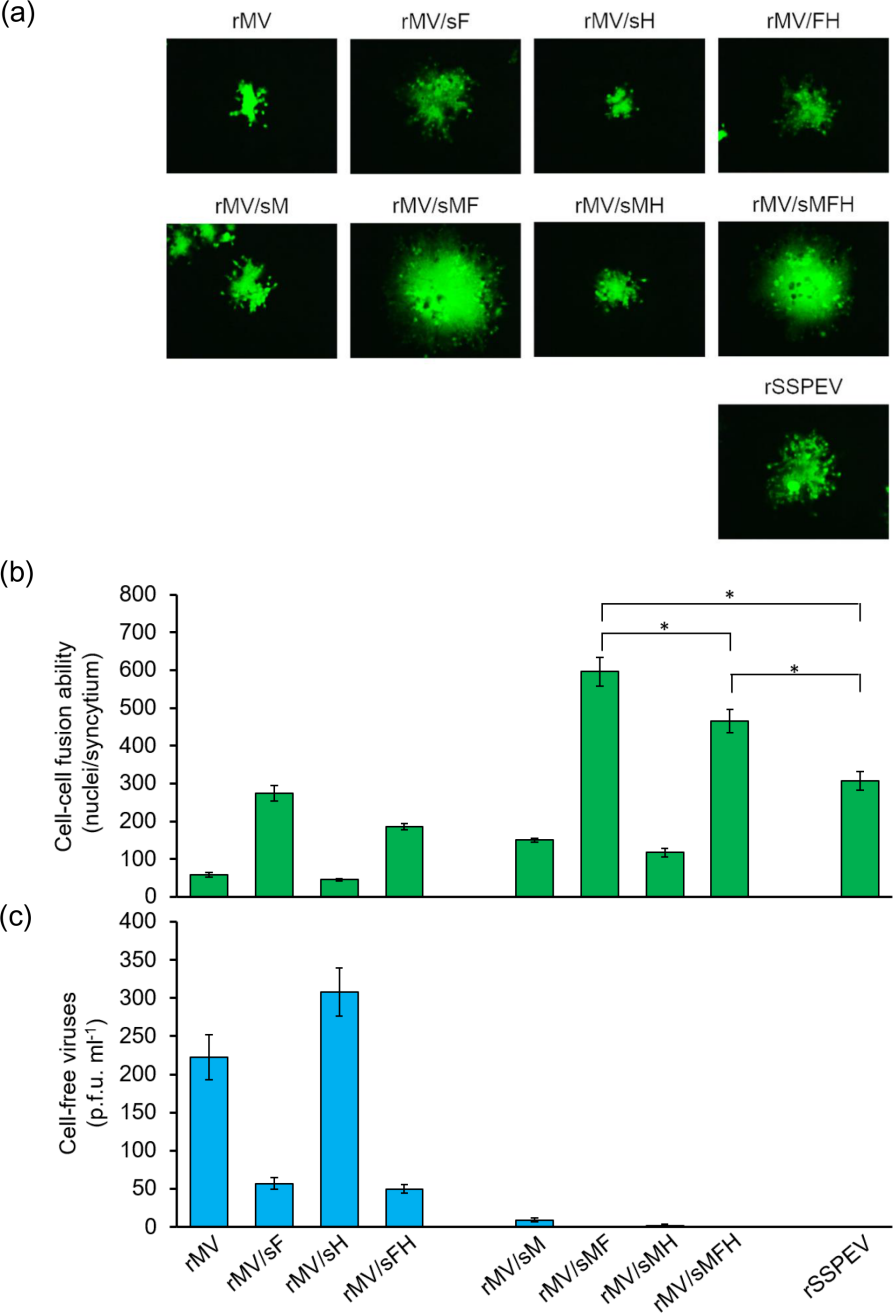
Growth characteristics of chimeric viruses. (**a**) Syncytium formation by chimeric viruses in Vero/hSLAM cells. Vero/hSLAM cells were infected with each chimeric virus in Fig. 1 at an m.o.i. of 0.001 and incubated at 37 °C. At 36 h p.i., EGFP-expressing infected cells were observed under a fluorescence microscope. Magniﬁcation, ×200. (**b**) Quantification of cell–cell fusion. Infected Vero/hSLAM cells shown in (**a**) were fixed, permeabilized and incubated with Hoechst 33 342. Cell–cell fusion is displayed as the number of nuclei in an EGFP-expressing syncytium. Nuclei in five syncytia were counted under a fluorescence microscope and are presented as average values with standard deviations (mean±sd). Statistical analysis was performed by unpaired Student’s *t*-test: *, *P*<0.05. (**c**) Infectious cell-free virus production by the chimeric viruses. B95a cells were infected with virus at an m.o.i. of 0.001. After incubation at 37 °C for 4 days, viruses in the culture fluid were titrated in Vero/hSLAM cells. Data from five independent experiments are presented as mean±sd.

The neurovirulence of some SSPE viruses correlates with elevated viral cell–cell fusion ability acquired from mutation(s) in the F protein, conferring hyperfusogenicity on the F protein [31, 32, 34]. Neurotoxicity of the SSPE virus Kobe-1 strain, however, cannot be achieved simply by mutation(s) in the F protein but requires additional mutation(s) in the M protein. Therefore, the contribution of these proteins of the Kobe-1 strain to viral fusion was evaluated in Vero/SLAM cells. Fig. 2a shows a typical syncytium formed by each chimeric virus (Fig. 1a), and Fig. 2b indicates the fusion ability estimated by enumerating nuclei. The higher fusion ability of rMV/sF compared to rMV demonstrates that the F protein of the Kobe-1 strain [F/SSPEV(Kobe) protein] acquired increased fusion activity compared with that of the MV ICB strain [F/MV(ICB) protein]. In contrast, the H protein of the Kobe-1 strain [H/SSPEV(Kobe) protein] slightly suppressed viral fusion (compare rMV/sH with rMV or rMV/sFH with rMV/sF). Replacement of the M protein of the MV ICB strain [M/MV(ICB) protein] by that of the SSPE Kobe-1 [M/SSPEV(Kobe) protein] enlarged the syncytia formed by rMV/sM, rMV/sMF, rMV/sMH, or rMV/sMFH, demonstrating the fusion upregulating activity of the M/SSPEV(Kobe) protein. rMV/sMF and rMV/sMFH, which had neurovirulence in mice (Fig. 1b), formed large syncytia. rMV/sMF showed greater fusion ability than rMV/sMFH, probably because of restriction of fusion by the H/SSPEV(Kobe) protein. rSSPEV demonstrated lower fusion ability than rMV/sMFH, indicating the involvement of viral element(s) other than the M, F and H proteins in viral fusion.

Infectious viral particle production by the chimeric viruses was also assayed (Fig. 2c). The M/SSPEV(Kobe) protein strongly suppressed release of infectious viral particles: trace amount by rMV/sM and rMV/sMH, or under the detectable amount by rMV/sMF and rMV/sMFH. By contrast, viruses carrying the M/MV(ICB) protein produced infectious virus, depending on the F protein. While rMV and rMV/sH had a high titre of cell-free infectious particles, rMV/sF and rMV/sFH [F protein was replaced by the F/SSPEV(Kobe) protein] showed limited release, an inverse correlation with cell–cell fusion ability. Therefore, the defect of the chimeric viruses in infectious particle production is caused by the M/SSPEV(Kobe) protein and in part by the F/SSPEV(Kobe) protein.

**Fig. 3.**
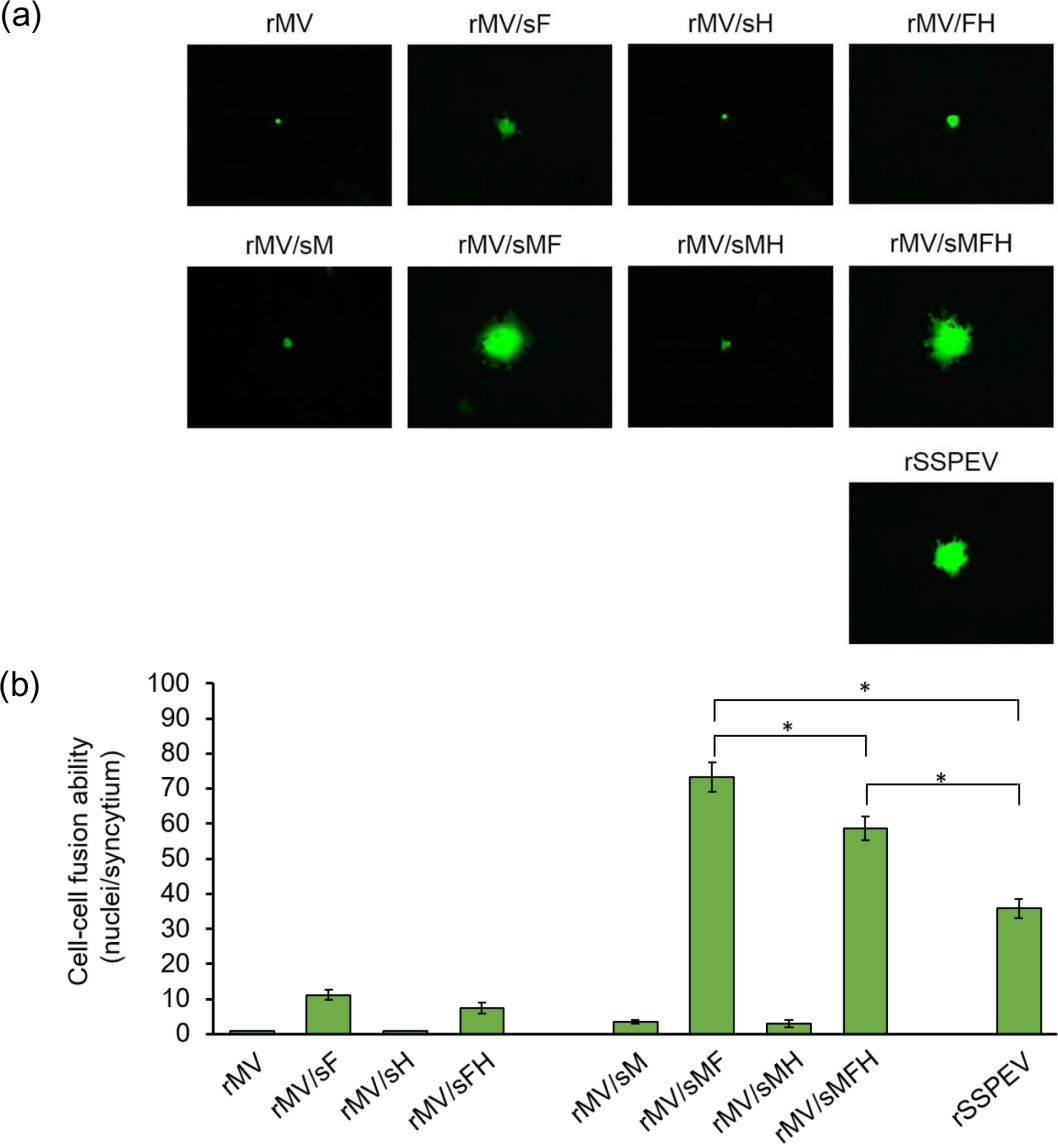
Enhanced cell–cell fusion by neurovirulent chimeric viruses in the receptor-negative Vero cells. (**a**) Syncytium formation by chimeric viruses in Vero cells. Vero cells were infected with the chimeric viruses in Fig. 1 at an m.o.i. of 0.001. At 72 h p.i., EGFP-expressing infected cells were observed under a fluorescence microscope. Magniﬁcation, ×200. (**b**) Quantification of Vero cell–cell fusion. Infected Vero cells shown in (**a**) were fixed, permeabilized and incubated with Hoechst 33 342. Nuclei in EGFP-expressing cells were enumerated under a fluorescence microscope. Cell–cell fusion is displayed as the number of nuclei in a syncytium. Data from five infected syncytia are presented as mean±sd. Statistical analysis was performed by unpaired Student’s *t*-test: *, *P*<0.05.

The highly fusogenic F proteins of SSPE viruses support spread of viral infection to receptor-negative cells by cell–cell fusion, a marker of neurovirulence enabling viral propagation in the brain [31, 32, 34], where the MV receptors, SLAM and nectin 4, are not expressed [9, 10]. Next, the ability of the chimeric viruses to induce cell–cell fusion was estimated in receptor-negative Vero cells. As shown in Fig. 3a, while rMV and rMV/sH did not spread, rMV/sF formed small syncytia. Next, the M/SSPEV(Kobe) protein was introduced to the chimeric viruses. While rMV/sM and rMV/sMH showed threefold increased fusion ability compared with rMV and rMV/sH, respectively, rMV/sMF and rMV/sMFH acquired six- to sevenfold increased fusion compared to rMV/sF and rMV/sFH, respectively (Fig. 3b). Therefore, the fusion activity of the F/SSPEV(Kobe) protein in Vero cells was significantly increased by the M/SSPEV(Kobe) protein. The neurovirulence of the chimeric viruses coincided with their fusion ability in Vero cells because only rMV/sMF, rMV/sMHF and rSSPEV (in that order) were neurovirulent (Fig. 1b). The results in Figs. 2 and 3 indicate that the fusion activity of the F/SSPEV(Kobe) protein, although higher than that of the F/MV(ICB) protein, is not sufficiently increased to support efficient growth of the virus in receptor-negative cells, and that the M/SSPEV(Kobe) protein compensates for the deficit. Viral neurovirulence paralleled the acceleration of viral cell–cell fusion and growth in receptor-negative cells in a manner that was dependent on both the F protein and the M protein.

### The M protein of the SSPE Kobe-1 strain lost its fusion-suppressing activity

**Fig. 4.**
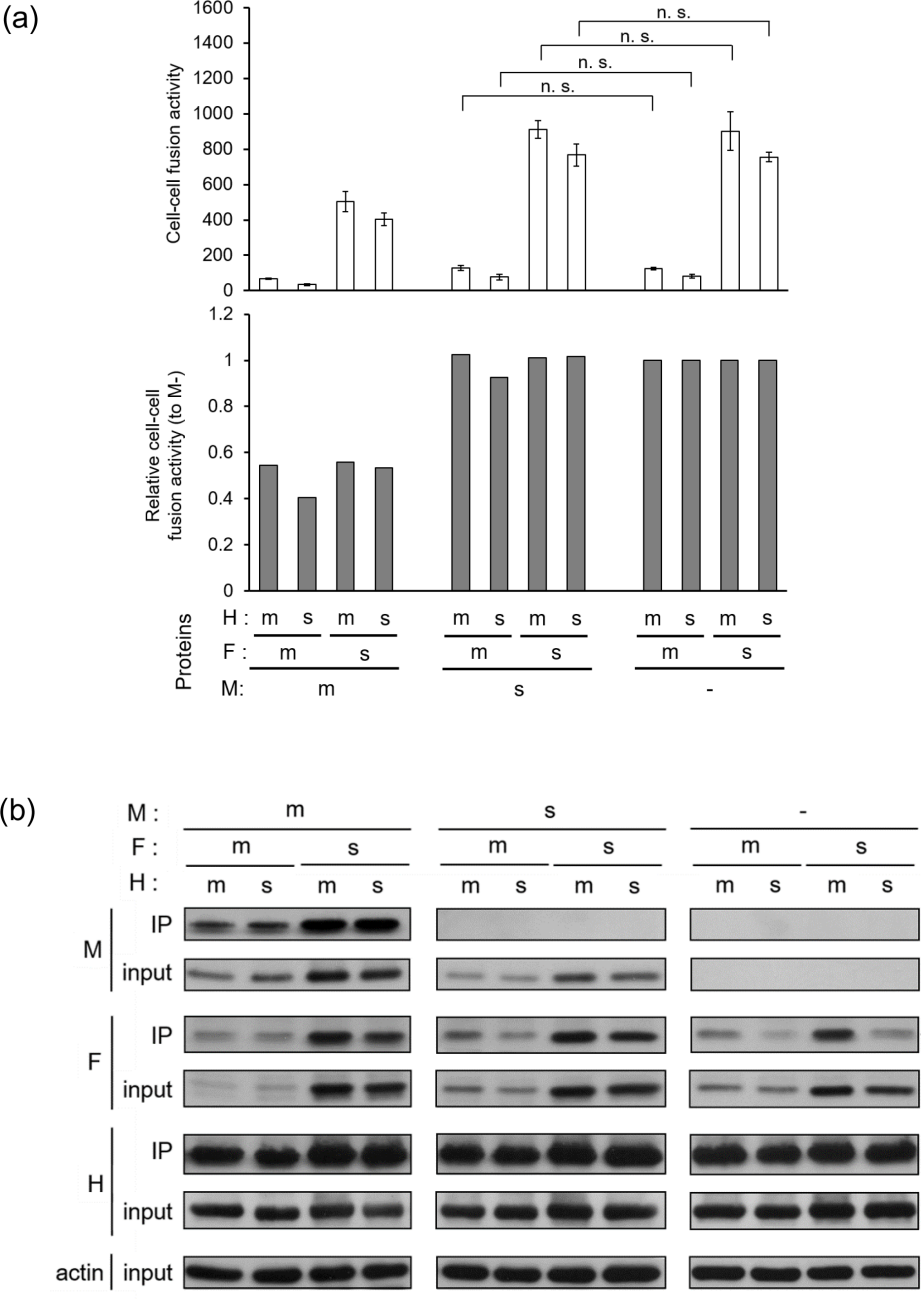
Cell–cell fusion suppression by the M protein of wild-type MV, which is abolished by the M protein of SSPE virus Kobe-1 strain. (**a**) Cell–cell fusion by H and F proteins co-expressed with the M protein. Vero/hSLAM cells were transfected with plasmids expressing myc-tagged H and F proteins of the MV ICB strain (**m**) or the SSPE virus Kobe-1 strain (**s**) together with that expressing the M protein of each virus or pCA7 vector for the without-M-protein condition (−). After incubation at 37 °C for 15 h, the cells were fixed and stained with crystal violet, and syncytia and nuclei per syncytium were enumerated under a microscope. Cell–cell fusion activity is shown as the product of two values and relative cell–cell fusion activity was calculated as described in the Methods section. *n*=3, mean±sd. Statistical analysis was performed by unpaired Student’s *t*-test: ns, not significant. (**b**) Interaction of M protein with the H/F protein complex. 293 T cells were transfected with plasmids expressing the myc-tagged H and F proteins of the MV ICB strain (**m**) or the SSPE virus Kobe-1 strain (**s**) together with that expressing the M protein of each virus or pCA7 vector as the control (−) and incubated at 37 °C. The cells were lysed 48 h after transfection and the lysates (input) were subjected to immunoprecipitation (IP) with an anti-myc antibody, followed by Western blotting using anti-myc, anti-F, anti-M and anti-β-actin primary antibodies, as described in the Methods section.

How does the M/SSPEV(Kobe) protein upregulate the fusion activity of the F protein? To answer this question, using a protein expression system, we investigated the behaviour of the M protein while the H/F protein complex executes cell–cell fusion. First, the fusion activity of the F/MV(ICB) protein and the F/SSPEV(Kobe) protein co-expressed with the H/MV(ICB) or H/SSPEV(Kobe) protein was estimated in the absence of the M protein. The F/SSPEV(Kobe) protein exhibited greater fusion activity than the F/MV(ICB) protein (Fig. 4a, right). The fusion activities were slightly lower when expressed with the H/SSPEV(Kobe) protein than with the H/MV(ICB) protein. The additionally expressed M/MV(ICB) protein suppressed the fusion activities of the F proteins by half those in the absence of the M protein for all combinations of F and H proteins (Fig. 4a, left). In the presence of the M/SSPEV(Kobe) protein, the fusion activities of the F proteins were similar to those in the absence of the M protein, indicating abolition of the suppressive effect by the M/MV(ICB) protein (Fig. 4a, middle). The high fusion activity of the F/SSPEV(Kobe) protein in the presence of the M/SSPEV(Kobe) protein is consistent with the high fusion ability of the neurovirulent viruses, rMV/sMF and rMV/sMFH, which carry the F and M proteins of the SSPE virus Kobe-1 strain.

The M protein of MV plays an important role in transporting H and F proteins on the surface of infected cells into the viral particle by binding to their cytoplasmic tail, interfering with cell–cell fusion of infected cells with adjacent cells [18]. To explore the molecular mechanism by which the M/SSPEV(Kobe) protein abolished the suppressive effect of the M/MV(ICB) protein on the fusion activity of the F protein, the interaction of the M protein with the H/F protein complex was investigated. Pulldown of the H protein precipitated the F protein, verifying the formation of a complex by the two proteins (Fig. 4b, right). When the M/MV(ICB) protein was co-expressed with the F and H proteins, the M protein co-precipitated with the H/F protein complex, confirming the interaction of the M/MV(ICB) protein with the H/F protein complex (Fig. 4b, left). The co-expressed M/SSPEV(Kobe) protein was not detected in the precipitate of the H/F protein complex (Fig. 4b, middle). Therefore, the M/SSPEV(Kobe) protein lost its ability to bind to the H/F protein complex, explaining the enhanced cell–cell fusion activity in the presence of the M/SSPEV(Kobe) protein. The M/SSPEV(Kobe) protein has no effect on the fusion activity of the F protein, resulting in abolition of the fusion-suppressing activity of the M/MV(ICB) protein.

The F/SSPEV(Kobe) protein has a shortened cytoplasmic tail, a feature typical of many SSPE viruses [25, 27, 35], but the interaction of the H/F protein complex composed of the F/SSPEV(Kobe) protein with the M/MV(ICB) protein was not impaired (Fig. 4b, left). If the H/F protein complex is transported into rMV/sF and rMV/sFH particles by the M/MV(ICB) protein, the infectivity of the released particles would not be reduced compared with that of rMV and rMV/sH. It is possible that the F/SSPEV(Kobe) protein induces cell–cell fusion before viral particle release due to the higher fusion activity than the F/MV(ICB) protein (Fig. 2c).

### The M protein of the MV ICB strain stabilizes the F protein

**Fig. 5.**
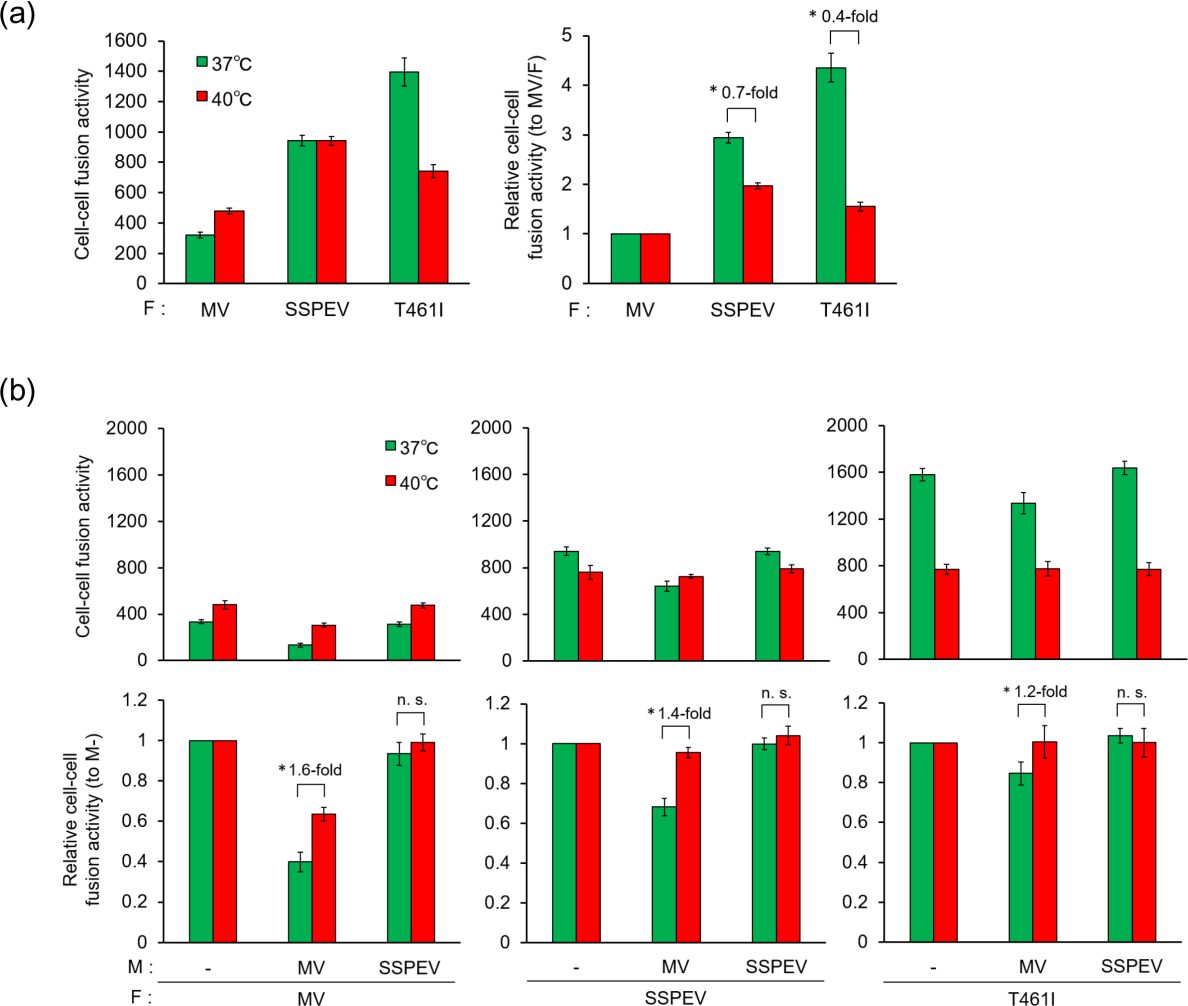
Stabilization of the F protein by the M protein of wild-type MV, which is abolished by the M protein of the SSPE virus Kobe-1 strain. (**a**) Temperature dependence of the cell–cell fusion activity of F protein. Vero cells were infected with vTF7-3 and transfected with a plasmid expressing the F protein of the MV ICB strain (MV) or the SSPE virus Kobe-1 strain (SSPEV), or carrying the T461I mutation (T461I), together with a plasmid expressing the myc-tagged H protein of the MV ICB strain. After incubation at 30 °C for 24 h, the cells were overlaid with Vero/hSLAM cells in the presence of 100 µM cycloheximide and incubated at 37 °C or 40 °C for 8 h. Cell–cell fusion activity and relative cell–cell fusion activity were determined as described in the Methods section. *n*=3. Data are mean±sd. Statistical analysis was performed by unpaired Student’s *t*-test: *, *P*<0.05. (**b**) Effect of the M protein on the thermodynamic stability of the F protein. Cell–cell fusion activity of F protein was determined as in (**a**) with co-transfection of a plasmid expressing the M protein of the MV ICB strain or the SSPE virus Kobe-1 strain, or the pcDNA3 vector. Effect of the M protein on F protein stability was estimated by normalizing the values in the presence of each M protein (M: MV, SSPEV) to that in their absence (M: −) at each temperature. *n*=3, mean±sd. Statistical analysis was performed by unpaired Student’s *t*-test: *, P＜0.05; ns, not significant.

MV F protein induces membrane fusion by a conformational change from the prefusion to the postfusion form, which is regulated by the stability of the prefusion molecule [36–38]. We estimated the fusion activity of the F protein in the presence of the M protein at 40 °C to examine the effect of the M protein on the stability of the F protein. The F protein with the T461I mutation (F/T461I) was included for comparison, because the T461I mutation in some SSPE viruses causes hyperfusogenicity and, therefore, neurovirulence in rodents [31, 39]. At 37 °C in the absence of the M protein, the F/SSPEV(Kobe) protein showed higher fusion activity than the F/MV(ICB) protein, but the F/T461I protein demonstrated the highest activity (Fig. 5a, left). Because the fusion activities of the F/SSPEV(Kobe) and F/T461I proteins relative to that of the F/MV(ICB) protein were lower at 40 °C than at 37 °C, these two F proteins were destabilized compared with the F/MV(ICB) protein (Fig. 5a, right). The smaller ratio (0.4-fold) between the values of the F/T461I protein at 37 °C and at 40 °C compared to that of the F/SSPEV(Kobe) protein indicated that the F/T461I protein is more destabilized than the F/SSPEV(Kobe) protein. Next, fusion activity was assayed in the presence of the M protein at 37 and 40 °C (Fig. 5b, upper panels) and evaluated as the relative fusion activity, setting the value of each F protein in the absence of the M protein at 1 (Fig. 5b, lower panels). When the M/MV(ICB) protein was co-expressed, the relative fusion activity of each F protein at 40 °C increased compared with that at 37 °C, indicating that the F protein was stabilized by the M/MV(ICB) protein. The magnitudes of the increases – 1.6-fold for the F/MV(ICB) protein, 1.4-fold for the F/SSPEV(Kobe) protein and 1.2-fold for the F/T461I protein – showed that the stabilizing effect of the M/MV(ICB) protein on the F protein correlated with the initial stability of the F protein (Fig. 5a, right). The F/SSPEV(Kobe) protein is stabilized more strongly than the F/T461I protein by the M/MV(ICB) protein, suppressing the fusion activity of the F/SSPEV(Kobe) protein. By contrast, no difference was found in the relative fusion activities at 37 and 40 °C with co-expression of the M/SSPEV(Kobe) protein, indicating that the M/SSPEV(Kobe) protein did not affect the stability of the F protein.

## Discussion

SSPE viruses are very rarely isolated from the brain of patients with SSPE. Genetically, the most striking feature of the isolates is the numerous mutations, including the frequent adenine-to-guanine or uracil-to-cytosine biased hypermutation, which often occur, especially around the M gene [25, 40–44]. The M protein of MV plays a central role in producing infectious viral particles by constituting their structure and by binding to the cytoplasmic tail of the H/F protein complex as well as to the viral RNP to transport them into the particle. The highly accumulated mutations around the M gene of SSPE viruses should impair M protein function [44–50]. The recombinant MVs whose M gene was replaced by that of an SSPE virus or was deleted from the genome demonstrated defects in the release of infectious viral particles and enhanced cell–cell fusion ability, showing that loss of M protein function switches the mode of propagation of MV. These highly assembly-defective fusogenic recombinant MVs slowed viral spread in the brain, penetrated more deeply into the brain parenchyma, and caused a prolonged chronic CNS infection, resulting in delayed death in mice compared to the parental MV [29, 30]. This observation differed from the fact that SSPE viruses are highly lethal and drive the infected rodents promptly to death [31, 32]. Therefore, these experiments did not prove the functionally defective M protein to be responsible for neurovirulence, although it may play a role in establishing persistent infection in the brain. In contrast, Ayata *et al*. for the first time reported that a recombinant MV (ICB strain) whose F gene was replaced by that of the SSPE Osaka-2 strain demonstrated neurovirulence in hamsters and that the T461I mutation in the F protein was responsible for pathogenicity [31]. In the same recombination experiment, the ICB strain acquired no neurovirulence from exchange of the M protein with that of the Osaka-2 strain. The F protein performs membrane fusion via a conformational change from the metastable prefusion form to the stable postfusion form to initiate infection by introducing RNP into target cells. It is generally accepted that mutations in the F protein destabilizing the prefusion form confer hyperfusogenicity on the F protein and increase cell–cell fusion and propagation in the brain, transforming an MV into a neurotoxic SSPE virus [32, 34, 39, 51–55]. The chimeric MV ICB strain, rMV/sF, whose F gene was exchanged for that of the SSPE Kobe-1 strain, however, did not display neurovirulence and at first we did not find the F gene to be involved in pathogenicity of the Kobe-1 strain. However, the chimeric MV, rMV/sMF, whose M and F genes were replaced by those of the SSPE Kobe-1 strain, was lethal in mice. In the SSPE Kobe-1 strain, the F and M proteins synergistically function in neurovirulence. To our knowledge, this is the first report of the involvement of the M protein in SSPE virus neurovirulence. We identified the G301W mutation in the F protein as destabilizing the F protein in the presence of M/SSPEV(Kobe) protein (manuscript in preparation).

Our results demonstrate that the M protein of the wild-type MV, M/MV(ICB) protein, interferes with the fusion activity of the F protein by binding to the H/F protein complex (Fig. 4). The neuropathogenicity of a recombinant MV ICB strain possessing a single amino acid substitution, T461I in the F protein [31, 34], could be explained by the fusion activity of the hyperfusogenic F/T461I protein even with the M/MV(ICB) protein, because it is too labile to be stabilized by M/MV(ICB) protein. The F protein of the SSPE virus Kobe-1 strain, F/SSPEV(Kobe) protein, is more stable than the hyperfusogenic F/T461I protein, although it is actually less stable and shows higher fusion activity than the wild-type F/MV(ICB) protein (Fig. 5a). The moderately destabilized F/SSPEV(Kobe) protein was stabilized by the M/MV(ICB) protein (Fig. 5b), which suppresses cell–cell fusion of the chimeric virus rMV/sF and interferes with its spread in the brain. Thus, rMV/sF did not exhibit neurovirulence. By contrast, the F/SSPEV(Kobe) protein in the chimeric virus, rMV/sMF, retained fusion activity in the presence of the M/SSPEV(Kobe) protein because it had lost its ability to stabilize F protein, allowing rMV/sMF propagation in the brain and neurovirulence. It might be desirable to introduce the M gene and F genes of an SSPE virus into wild-type MV to investigate pathogenicity without the confounding effect of the wild-type MV M protein.

While MV persists in the brain by escaping from the immune system, mutant viruses possessing a defect in viral particle release may have a selection advantage [56, 57]. The results in Fig. 2 demonstrate that infectious particle release related inversely to cell–cell fusion ability: the lower the particle release, the greater the cell–cell fusion. The M/SSPEV(Kobe) protein was not transported to the plasma membrane and was localized throughout the cytosol [23]. The absence of the M protein in the plasma membrane might explain not only the limited particle release but also the increased cell–cell fusion. This is because the F protein-stabilizing effect of the M protein was abolished by the lack of interaction with the H/F protein complex in the plasma membrane (Fig. 4b). The M protein of the SSPE Biken strain was detected in the soluble cytosolic fraction but not in the plasma membrane fraction [49]. If SSPE viruses selected in the brain as particleless viruses carry the M protein of the aberrant transport character, such an M protein may enhance the cell–cell fusion of SSPE viruses.

Although SSPE has a very low frequency, around 1 case per 100 000 cases of measles [58–60], more recent studies have estimated a higher risk of 22 : 100,000 [61], and 1 : 1367 or 1 : 1700 to 1 : 3300 in children who acquired measles under the age of 5 years [62, 63]. Like the T461I mutation, mutations in the F protein in the domains responsible for stability and conformational change, the HR-B domain in the stalk region near the junction with the head region (amino acid 456–495) or the DIII domain in the head region (amino acid 53–296) [17, 36, 64], confer neuropathogenicity, leading to SSPE. The probability, however, of those mutations is not sufficient to explain the high frequency of SSPE. During persistent infection, if a particleless SSPE virus carries transport-defective M protein, the M protein will not suppress cell–cell fusion. Such M proteins will ensure sufficient cell–cell fusion for growth in the brain of virus with a moderately destabilized F protein, like the F/SSPEV(Kobe) protein, increasing the likelihood of SSPE. Investigation of the cooperative roles of the F and M proteins in neuropathogenicity is underway with SSPE strains other than Kobe-1.

## Methods

### Cells and viruses

Vero cells constitutively expressing human SLAM (Vero/hSLAM) (a gift from Y. Yanagi, Kyushu University) [4] and Vero cells were maintained in Dulbecco’s modified Eagle’s medium (DMEM) supplemented with 8 % foetal bovine serum (FBS). 293 T cells and cells of a marmoset B-cell line transformed with Epstein–Barr virus (B95a) were maintained in high-glucose DMEM supplemented with 10 % FBS. SH-SY5Y human neuroblastoma cells were maintained in high-glucose DMEM supplemented with 10 % FBS and 1 % MEM non-essential amino acid solution (Fujifilm Wako Pure Chemical Corporation, Osaka, Japan). BHK cells constitutively expressing T7 RNA polymerase (BHK/T7-9) (a gift from N. Ito and M. Sugiyama, Gifu University) [65] were maintained in RPMI 1640 medium supplemented with 8 % FBS and 0.6 mg ml^−1^ hygromycin B. Isolation of SSPE virus Kobe-1 strain was described previously [33]. Recombinant MVs (rMVs) were generated according to Seki *et al*. [66] as described previously [67] using MV full-length genome plasmids. T7 RNA polymerase-expressing vaccinia virus (vTF7-3) [68] was a gift from B. Moss (National Institutes of Health, USA).

### Plasmid construction

The cDNA of the genome of the SSPE virus Kobe-1 strain (GenBank: AB254456) was synthesized by reverse-transcription PCR. The SalI-SacII fragment [nucleotides (nt) 3365–4921 according to the ICB strain genome sequence (GenBank: AB016162)] of the p(+)MV323c72-EGFP plasmid [67] derived from the full-length genome plasmid of the MV ICB strain, p(+)MV323-EGFP (a gift from Y. Yanagi) [69], was replaced by the corresponding region of the Kobe-1 strain, generating a plasmid with the full-length genome of the ICB strain carrying the M gene of the SSPE virus Kobe-1 strain [p(+)MV323-EGFP/sM]. The SacII-PacI fragments (nt 4922–7243) of p(+)MV323c72-EGFP and p(+)MV323-EGFP/sM were replaced by the corresponding fragment of the Kobe-1 strain, which generated p(+)MV323-EGFP/sF carrying the F gene of the Kobe-1 strain and p(+)MV323-EGFP/sMF carrying the M and F genes of the Kobe-1 strain, respectively. To exchange the ICB strain H gene for that of the Kobe-1 strain, the PacI-SpeI fragments (nt 7243–9175) of p(+)MV323c72-EGFP, p(+)MV323-EGFP/sM, p(+)MV323-EGFP/sF and p(+)MV323-EGFP/sMF were replaced by the corresponding fragment of the Kobe-1 strain, yielding p(+)MV323-EGFP/sH, p(+)MV323-EGFP/sMH, p(+)MV323-EGFP/sFH and p(+)MV323-EGFP/sMFH, respectively. The plasmid p(+)MV323-EGFP/SSPEV containing all genes of the Kobe-1 strain was constructed by replacing the SmaI-SalI fragment (nt 839–3364) and the SpeI-Eco47III fragment (nt 9176–15767) of p(+)MV323-EGFP/sMFH with the corresponding regions of the Kobe-1 strain.

For protein expression, the M gene (nt 3438–4445), the F gene (nt 5458 to 7110) and the H gene (nt 7271 to 9124) were amplified by PCR using p(＋)MV323c72-EGFP or the cDNA of the SSPE virus Kobe-1 strain as the template. The M and F genes were cloned into the pCA7 vector or pcDNA3 vector (Invitrogen, Carlsbad, CA, USA), generating pCA7-M/MV(ICB), pCA7-M/SSPEV(Kobe), pCA7-F/MV(ICB), pCA7-F/SSPEV(Kobe), pcDNA-M/MV(ICB), pcDNA-M/SSPEV(Kobe), pcDNA-F/MV(ICB) and pcDNA-F/SSPEV(Kobe), respectively. The amplified H genes were cloned into the pCA7-myc vector or pcDNA3-myc vector prepared by inserting the myc-tag fragment downstream of the multiple cloning site of the pCA7 and pcDNA3 vectors, generating pCA7-H-myc/MV(ICB), pCA7-H-myc/SSPEV(Kobe), pcDNA-H-myc/MV(ICB) and pcDNA-H-myc/SSPEV(Kobe), respectively. To introduce the Thr to Ile mutation at residue 461 (T461I) of the F protein, the ICB F gene carrying a C to T substitution at nt 6839 was cloned into the pcDNA3 vector.

### Virus titration

Monolayers of Vero/hSLAM cells in 24-well plates were infected with serially diluted virus samples. After 1 h of incubation at 37 °C, the virus samples were removed, and the cells were overlaid with DMEM containing 8 % FBS and 2.5 % methylcellulose. After 2 days, plaque-forming units (p.f.u.) were determined by enumerating plaques expressing EGFP under a fluorescence microscope (Axioskop; Zeiss, Jena, Germany).

### Virus challenge

BALB/c suckling mice purchased from CLEA Japan, Inc. (Tokyo, Japan) were used prior to 3 weeks old after passing a medical inspection. Mice were under anaesthesia when inoculated intracerebrally with 7×10^2^ p.f.u. of each recombinant chimeric virus in a 20 µl suspension of B95a cells. After inoculation, clinical symptoms were observed daily, and moribund mice were euthanized.

### Cell–cell fusion assay (syncytium formation by recombinant viruses)

Vero cells or Vero/hSLAM cells cultured in 24-well plates were infected with rMVs at a multiplicity of infection (m.o.i.) of 0.001 and incubated at 37 °C for 36 or 72 h. The cells were fixed in 1 % paraformaldehyde, permeabilized with 1 % Triton X-100, and stained with Hoechst 33 342 (Sigma-Aldrich, St Louis, MO, USA). The number of nuclei in an EGFP-expressing syncytium was counted under a fluorescence microscope.

### Cell–cell fusion assay (syncytium formation by expressed F, H and M proteins)

Subconfluent monolayer cultures of Vero/hSLAM cells in 24-well plates were transfected with 0.5 µg of the F protein-expressing plasmid [pCA7-F/MV(ICB) or pCA7-F/SSPEV(Kobe)] and 0.5 µg of the H protein-expressing plasmid [pCA7-H-myc/MV(ICB) or pCA7-H-myc/SSPEV(Kobe)] together with 1 µg of the M protein-expressing plasmid [pCA7-M/MV(ICB) or pCA7-M/SSPEV(Kobe)] or pCA7 vector and incubated at 37 °C. At 15 h post-transfection, the cells were fixed and stained with crystal violet, and syncytia and nuclei per syncytium were enumerated under a microscope. Cell–cell fusion activity was shown as the product by multiplying the two values. To estimate the effect of the M protein, relative cell–cell fusion activity was calculated by dividing the cell–cell fusion activity of each combination of F and H proteins in the presence of M protein by that in the absence of the M protein.

### Immunoprecipitation and Western blot analyses

Subconfluent monolayer cultures of 293 T cells in six-well plates were transfected with 0.5 µg of the F protein-expressing plasmid [pCA7-F/MV(ICB) or pCA7-F/SSPEV(Kobe)] and 0.5 µg of the H protein-expressing plasmid [pCA7-H-myc/MV(ICB) or pCA7-H-myc/SSPEV(Kobe)] together with 1 µg of the M protein-expressing plasmid [pCA7-M/MV(ICB) or pCA7-M/SSPEV(Kobe)] or pCA7 vector and incubated at 37 °C. At 48 h post-transfection, cells were suspended in 0.1 M HEPES/NaOH (pH 7.5) containing cross-linking reagent dithiobis (succinimidyl propionate) (DSP; Thermo Fisher Scientific, Waltham, MA, USA) followed by incubation for 2 h, at 4 °C, and the cross-linking reaction was stopped by 10 mM Tris/HCl (pH 7.5). The cells were solubilized with 1 ml of lysis buffer consisting of 1.0 % Triton X-100, 10 mM Tris/HCl (pH 7.5) and 5 mM NaCl [23]. The cell extracts were subjected to immunoprecipitation after centrifugation at 13 000 ***g*** for 10 min at 4 °C.

A small amount (28 µl) of cell extract was mixed with SDS loading buffer as a total cell extract sample. The rest of the extract was incubated for 1 h, at 4 °C, with protein G-conjugated magnetic beads (Bio-Rad, Hercules, CA, USA), which had been preincubated with an anti-myc rabbit polyclonal antibody (Cell Signaling Technology, Danvers, MA, USA) for 1 h at room temperature. Immune complexes were obtained by magnetization and washed with lysis buffer according to the instructions for the magnetic beads, and the magnetized proteins and total cell extracts were subjected to SDS-PAGE in 10 % polyacrylamide gels followed by electroblotting onto PVDF membranes. Proteins were detected by incubating the membranes with a mouse monoclonal antibody against MV M protein (Merck Millipore, Burlington, MA, USA), rabbit polyclonal antibody against MV F protein [70], rabbit polyclonal antibody against myc-tag (GeneTex, Irvine, TX, USA), or mouse monoclonal antibody against β-actin (Cell Signaling Technology), followed by incubation with an HRP-conjugated goat anti-mouse IgG (Santa Cruz Biotechnology, Dallas, TX, USA), or goat anti-rabbit IgG (Cell Signaling Technology) secondary antibody. Proteins were visualized using the ECL Plus Western blotting detection system (GE Healthcare, Chicago, IL, USA) by exposure to an autoradiography film.

### Evaluation of F protein stability

Thermodynamic stability of F protein was evaluated based on the temperature dependence of the cell–cell fusion activity relative to that of standard F protein, as reported previously [70–72]. The relative cell–cell fusion activity of F protein was calculated by dividing the cell–cell fusion activity at 37 °C or 40 °C by that of the F/MV(ICB) protein at the same temperature. The effect of M protein on the thermodynamic stability of F protein was evaluated in the same way. The cell–cell fusion activity of the F protein in the presence of the M protein at 37 °C or 40 °C was divided by that in the absence of the M protein at the same temperature to estimate relative cell–cell fusion activity. F protein stability was assessed as follows: destabilized when relative cell–cell fusion activity was higher at 37 °C than at 40 °C, and stabilized when relative activity at 40 °C was higher than that at 37 °C.
